# The Book World of Medicine and Science

**Published:** 1912-01-06

**Authors:** 


### THE BOOK WORLD OF MEDICINE AND SCIENCE.

Infectious Diseases and Their Preventive Treatment. By E. C. Seaton, M.D. (Lond.), F.R.C.P. (London: University of London Press, Hodder and Stoughton. 1911. Pp. 213. Price 7s. 6d.)

A detailed review of this book is unnecessary, as it is itself only intended to give an idea of the ways and means by which systems, schemes, and plans recommended by leaders in the campaign against infectious diseases have proved feasible and effective. Dr. Seaton, as is well known, has had a long and important experience in public health matters, and as consulting medical officer to the Surrey County Council is himself one of the leaders in the field of preventive medicine. The volume practically reproduces the Chadwick Lectures for 1910, with some amplification. The subject of the science of administration is one which is too seldom discussed by those who have had actual experience, and for this reason the book should find a ready acceptance. The particular branch of the subject dealt with by Dr. Seaton is that concerned with notification, hospital isolation, and other distinctly medical preventive measures. The lectures contain many valuable suggestions, and much of interest is to be learnt from the lessons of past epidemiological experience, while the book is rendered extremely valuable by the inclusion of numerous unique charts and diagrams illustrating the prevalence and mortality of the principal infectious diseases during the last fifty years or so. We may specially call attention to the series of charts showing the secondary and return cases of scarlet fever in the Epsom and Dorking districts. The book is one that should appeal to every one engaged in fever work or, indeed, any of the branches of the public health service.

A Manual of Midwifery. By G. Balfour Marshall, M.D. (Glasgow: J. Maclehose and Sons. 1912. Pp. 401. Price 14s. net.)

Whenever, as in the present case, an author offers to the profession a manual which is avowedly for practitioners and students as well, he sets out to cater for two classes whose immediate interests are not quite the same. To the practitioner it is practical diagnosis and treatment which is all-important; to the student pathology, ætiology, and statistical information has an equal, and sometimes an even greater value, owing to the exigencies of examinations. With regard to obstetric medicine, however, this divergence of requirements is less than in most other departments of medical practice; for medical students are early initiated into the practical applications of obstetric principles, and they know that after qualification this subject is one in which they will have ample opportunities of proving their proficiency.

This volume is declared to be founded on the author's lecture notes; and indeed it bears many traces of such an origin which would have been better removed in the process of expansion into a book. Terseness is all very well for blackboard tabulation in a lecture theatre; but in cold print it is unlovely, and in this case often results in obscurity. Indeed this latter quality is far too much in evidence altogether, chiefly due to a very slipshod and careless method of writing. There is also a strong tendency to the use of singular verbs in agreement with plural nouns; and to chop about from singular to plural in the same sentence, or from the third person to the first. But this is not the worst: nor would it matter greatly if the meaning were always evident. Many sentences are so vaguely worded as to be actually of doubtful import. Thus one reads "Chorion-epithelioma most often follows this disease" (hydatidiform mole). There is nothing in the context to show whether the author means that chorion-epithelioma is the commonest sequela of hydatidiform mole; or that hydatidiform mole is the commonest precursor of chorion-epithelioma; or that chorion-epithelioma ensues in most cases of hydatidiform mole; or that the sequence is more rarely the reverse way. Then, too, pregnancy is alleged to last on the average 280 days from the date of the last menstrual period; presumably, the first day of the period is meant, but the author does not say so.

If any further examples of muddle are wanted, consider this sentence: "There are three clinically distinct conditions from which pregnant women may suffer. . . . These are albuminuria, due to pregnancy kidney, which may end in eclampsia, pernicious vomiting, and acute yellow atrophy." We defy anyone to distinguish the three clinically distinct conditions which the author has in mind; and it may be added that the description subsequently given of acute yellow atrophy of the liver is not at all an accurate one. Consider also: "In menstruation the uterine mucosa thickens two or three times." By this probably is meant that the mucosa grows to twice or thrice its original thickness during the intermenstrual period; but that is certainly not the face-value of the words quoted.

On matters of practice there are also some debatable views advanced. Thus episiotomy is advised when perineal rupture seems to be inevitable. Tonic contraction of the uterus is said to be caused by "too frequent examinations" (sc. vaginal). Infant feeding is not very satisfactorily discussed. An aseptic thrombosis of the veins of the leg is described; but no clear direction is given as to how long rest in bed is to be prescribed in the treatment of this condition. On the whole we are forced to conclude that both practitioners and students are already provided with better obstetrical text-books than this latest addition to the number.

Tropical Hygiene for Anglo-Indians and Indians. By Surgeon General C. P. Lukis, M.D., F.R.C.S., C.S.I. and Major R. J. Blackham, D.Ph., R.A.M.C. (Thacker, Spink and Co., 2 Creed Lane. 1911. Price 4s. 6d. net.)

We have read this little work with great interest. It seems to us that it fulfils a decided want, since it deals with elementary questions of tropical hygiene in a lucid and common-sense manner. The various tropical diseases are described clearly, without undue detail, such as would be more in consonance with the object of a text-book than a popular exposition such as this, the essentials of which are that every chapter should be easily understood by every lay reader. An admirable series of hints adds greatly to the value of the book. Matters such as hygiene of the person, housing in the tropics, diet, refuse disposal, and disinfection are considered in greater detail. The instruction and information given are everywhere sound, and generally conveyed in an interesting manner. The book deserves a place in the library of every man who goes into the tropics, and is specially recommended to the ship's surgeon, since it gives much valuable information that may be of use to him. It is a model of what such a work should be, and we congratulate the writers and the publishers on their respective shares in its production.

